# Endoscopic retrieval of a proximally migrated pancreatic stent utilizing a basket through a catheter in surgically altered anatomy: “Basket-through-the-catheter” technique

**DOI:** 10.1055/a-2512-7794

**Published:** 2025-01-28

**Authors:** Haruka Toyonaga, Takuya Takayama, Tatsuya Nakagawa, Masahiro Orino, Ryosuke Tonozuka, Takao Itoi, Masaaki Shimatani

**Affiliations:** 1 50196 Department of Gastroenterology and Hepatology, Kansai Medical University Medical Center, Moriguchi, Japan; 2 13112 Department of Gastroenterology and Hepatology, Tokyo Medical University, Shinjuku-ku, Japan

Endoscopic retrieval of proximally migrated pancreatic duct stents (PDSs) is challenging due to the narrow and winding pancreatic duct (PD), the presence of PD stenosis, lack of optimal retrieval devices, and potential of severe adverse events. In cases with surgically altered anatomy, scope maneuverability is compromised, and device options are restricted. While various retrieval methods have been reported 1 2 3 4 5 , we successfully retrieved a migrated PDS employing the “basket-through-the-catheter” technique ( Fig. 1 , Video 1 ).

**Fig. 1:**
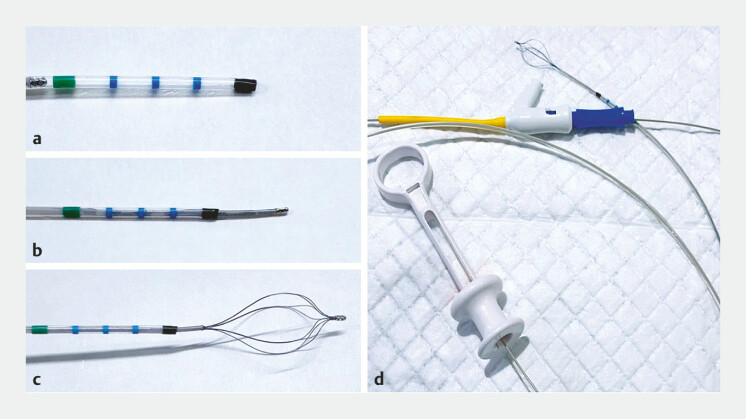
**a** A tapered tip of the endoscopic retrograde cholangiopancreatography (ERCP) catheter was pre-cut to allow the basket to emerge. **b, c** A fine basket designed for cholangiopancreatoscopy can be delivered through the catheter. **d** The basket-through-the-catheter technique enables the retrieval of stones or foreign bodies located beyond tight strictures or sharp angulations, where device delivery is challenging.

The basket-through-the-catheter technique enabled successful retrieval of a proximally migrated pancreatic duct stent in a patient with surgically altered anatomy, overcoming the challenges of limited balloon-enteroscopy maneuverability and ductal stenosis, and ultimately avoiding surgical intervention.Video 1

A 74-year-old man, with a history of pylorus-preserving pancreatoduodenectomy for duodenal ampullary carcinoma, had undergone balloon dilation and PDS placement to manage stenosis at the pancreatojejunostomy and pancreatic tail. The previously placed 5-Fr, 9-cm straight-type PDS had completely migrated into the PD, with the distal end embedded in a PD side branch ( Fig. 2 ). Additional dilation of the anastomotic and pancreatic duct strictures was performed, and multiple retrieval attempts were unsuccessful due to the stent’s distal end embedding, strictures, angulation in the PD, and limited space in the caudal PD ( Fig. 3 ).

**Fig. 2:**
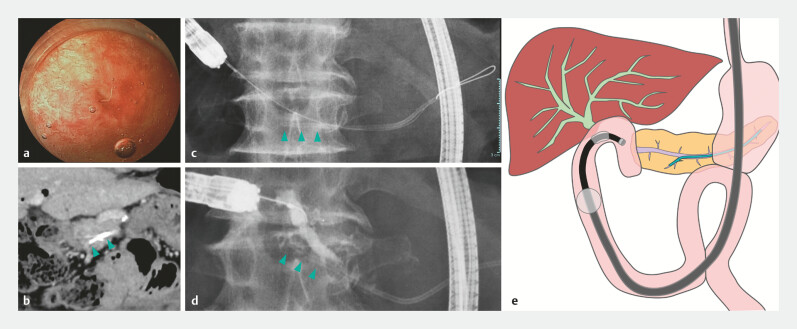
**a** Endoscopic image of pancreaticojejunal anastomotic stricture (PJS). The previously placed pancreatic duct stent (PDS) had migrated into the pancreatic duct (PD), and the PJS had restenosed. **b** Computed tomography image of the embedded distal end of the PDS (arrowhead) into the PD branch. **c, d** Fluoroscopic images of the proximally migrated PDS and the embedded distal end of the stent (arrowhead). **e** Schema of the PDS proximal migration.

**Fig. 3:**
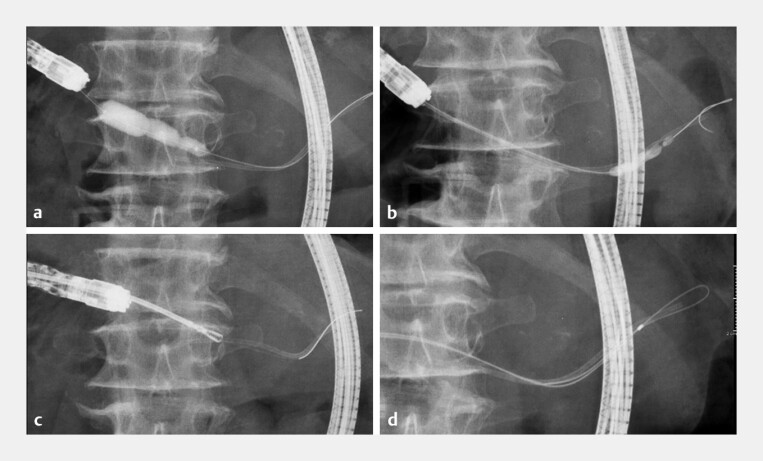
To retrieve the migrated stent, we attempted several techniques. These methods were unsuccessful due to the presence of PD strictures, sharp angulations, and the embedding of the stent within the PD branch. **a** Dilation of the PJS and PD strictures using a balloon dilator. **b** Pulling the stent out with an inflated balloon. **c** Attempted retrieval with forceps. **d** Attempted retrieval using a guidewire loop with a double-lumen catheter.

An endoscopic retrograde cholangiopancreatography (ERCP) catheter (G-Cannula; Gadelius Medical K.K., Tokyo, Japan) was advanced to the stent’s proximal end beyond the strictures, serving as a delivery sheath for a fine basket (SpyBasket; Boston Scientific, Marlborough, Massachusetts, USA) designed for cholangiopancreatoscopy. The basket-through-the-catheter technique was employed, with the catheter tip pre-cut to allow the basket to emerge and grasp the proximal end of the stent. Firm traction inverted and retrieved the stent from the PD ( Fig. 4 , Fig. 5 ). Aware of potential PD branch injury where the stent’s distal end was embedded, we proceeded cautiously, noting that the small diameter of the 5-Fr stent favored successful extraction with minimal complications. Surgical resection was considered an alternative, reinforcing our decision for endoscopic retrieval. The patient experienced only transient hyperamylasemia, and pancreatography confirmed the absence of residual stent fragments or pancreatic fistula.

**Fig. 4:**
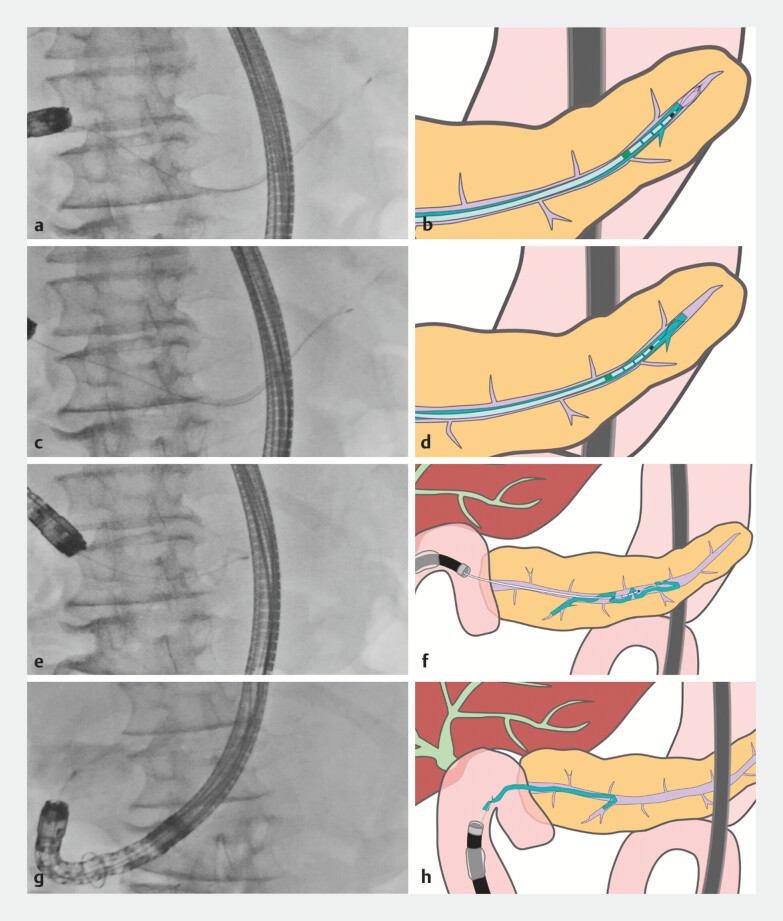
Fluoroscopic images and schema of the retrieval procedure using the basket-through-the-catheter technique. **a, b** The catheter was advanced deep into the PD beyond the proximal end (pancreatic tail side) of the stent, and a small-diameter basket was delivered through the catheter and deployed. **c, d** Despite the limited maneuverability in the deep PD, the end of the stent was successfully grasped with the basket through rotation and repeated advancement and retraction. **e, f** Fully aware of the risk that the distal end of the stent could damage the branch of the PD, and with no alternative methods available, we proceeded with stent retrieval using the basket-through-the-catheter technique. Given the small diameter of the 5-Fr diameter of the stent and the likelihood that it would bend and flip within the PD, we assessed that successful retrieval was achievable. **g, h** As anticipated, the PDS inverted within the duct, allowing us to pull the proximal end, grasped by the basket, from within the PD toward the intestinal side. We proceeded with cautious retrieval and successfully removed the entire stent, ensuring that no fragments were retained within the PD

**Fig. 5:**
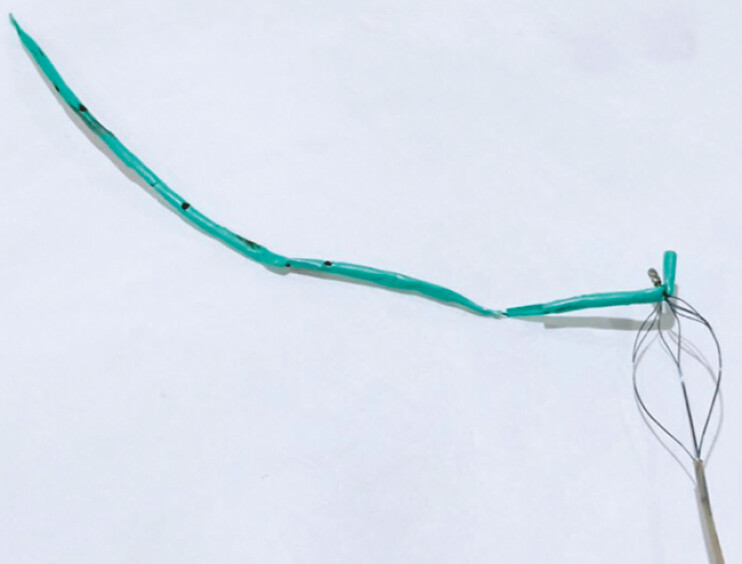
The retrieved stent showed that the basket had firmly grasped the tip of the stent.

Endoscopy_UCTN_Code_TTT_1AR_2AG

## Notes

Endoscopy E-Videos https://eref.thieme.de/e-videos
*E-Videos* is an open access online section of the journal *Endoscopy* , reporting on interesting cases and new techniques in gastroenterological endoscopy. All papers include a high-quality video and are published with a Creative Commons CC-BY license. Endoscopy E-Videos qualify for HINARI discounts and waivers and eligibility is automatically checked during the submission process. We grant 100% waivers to articles whose corresponding authors are based in Group A countries and 50% waivers to those who are based in Group B countries as classified by Research4Life (see: https://www.research4life.org/access/eligibility/ ). This section has its own submission website at https://mc.manuscriptcentral.com/e-videos .
